# Diversity of Bacteria and Archaea in hypersaline sediment from Death Valley National Park, California

**DOI:** 10.1002/mbo3.20

**Published:** 2012-06

**Authors:** Jong-Shik Kim, Mfundi Makama, Janine Petito, Nyun-Ho Park, Frederick M Cohan, Robert S Dungan

**Affiliations:** 1 Gyeongbuk Institute for Marine Bioindustry688-3 Hoojung-Ri, Jukbyeon-Myeon, Uljin-Gun, Gyeongsangbuk-Do, 767-813, Republic of Korea; 2 Department of Biology, Wesleyan UniversityMiddletown, Connecticut 06459; 3 Northwest Irrigation and Soils Research Laboratory,, Agricultural Research Service, U.S. Department of Agriculture3793 North 3600 East, Kimberly, Idaho 83341

**Keywords:** Archaea, Bacteria, Ecotype Simulation, hypersaline sediment, phylogeny, 16S rRNA gene

## Abstract

The objective of this study was to phylogenetically analyze microorganisms from the domains Bacteria and Archaea in hypersaline sediment from Death Valley National Park. Using domain-specific primers, a region of the 16S rRNA gene was amplified using polymerase chain reaction (PCR), and the product was subsequently used to create a clone library. A total of 243 bacterial clones, 99 archaeal clones, and 209 bacterial isolates were examined. The 243 clones from Bacteria were affiliated with the following groups: the Bacilli (59 clones) and Clostridia (1) of the Firmicutes, Bacteroidetes (90), Proteobacteria (27), Cyanobacteria (18), Gemmatimonadetes (41), candidate division OP1 (5), Actinobacteria (1), and the Deinococcus-Thermus division (1). Within the class Bacilli, 46 of 59 clones were tentatively identified as 10 unclassified species. The majority of bacterial isolates (130 of 209) were more closely related to the *Bacillus subtilis–B. licheniformis* clade than to any other recognized taxon, and an Ecotype Simulation analysis of *B. subtilis* relatives identified four previously unknown ecotypes. Several new genera were discovered within the Bacteroidetes (4) and the Gemmatimonadetes (2). Of the 99 archaeal clones, 94 were tentatively identified as belonging to 3 new genera within the *Halobacteriaceae*; other clones represented novel species within each of 4 established genera.

## Introduction

Halophilic Bacteria and Archaea can survive in hypersaline environments where salt concentrations approach saturation. Some halophiles are economically important for industrial and biotechnological applications, and many new applications are currently being investigated (Oren 2010; Litchfield 2011; Pérez et al. 2011). While new advances have been slow, there is great interest in the use of halophilic microorganisms to degrade organic pollutants and to produce biopolymers, biosurfactants, and compatible solutes (Marhuenda-Egea and Bonete 2002; Le Bornge et al. 2008; Kebbouche-Gana et al. 2009). Thus, increasing our understanding of halophile diversity is an essential step toward discovery of new and potentially useful organisms.

Recent research on microorganisms in hypersaline environments has resulted in the discovery of several new species and genera of Bacteria and Archaea (Arahal et al. 1996; Eder et al. 2001; Antón et al. 2002; Yoon et al. 2002; Xu et al. 2007a; Chen et al. 2008). Most of the extreme halophiles are classified into the domain Archaea, with 28 genera in the *Halobacteriaceae* alone (Kamekura 1998). However, there are also some extremely and moderately halophilic Bacteria, such as *Salinibacter ruber* M31 of the Bacteroidetes, which is an extremely halophilic bacterium isolated from a solar saltern (Antón et al. 2002). Halophilic Bacteria and Archaea have been isolated from a variety of hypersaline environments such as saline soils (Chen et al. 2008), seas (Oren 1983), solar salterns (Yoon et al. 2002; Cui et al. 2007; Jeon et al. 2007), saline lakes (Lim et al. 2005; Ren and Zhou 2005), and ancient halite (Mormile et al. 2003; Schubert et al. 2010).

Death Valley National Park (DVNP) in California is the hottest and driest place in North America, with daily summer temperatures sometimes exceeding 49°C and has an average annual rainfall of 38 mm. As a result of the extreme climate and topography, evaporite crust covers large areas of the valley floor. Until recently, very few studies had characterized microorganisms in hypersaline environments of DVNP (Huang et al. 2000; Alexander and Imhoff 2006; Bardavid et al. 2007). Here, we have used a cultivation-free, polymerase chain reaction (PCR)-based approach to identify microorganisms from the domains Bacteria and Archaea from environmental DNA extracted from hypersaline sediment in DVNP; we have also cultivated isolates on various media. Using Bacteria*-* and Archaea-specific primers, a region of the 16S rRNA gene was amplified using PCR, and the products were subjected to phylogenetic and Ecotype Simulation analyses. The aim of this study was to increase the general understanding of microbial diversity in hypersaline environments.

**Figure 1:**
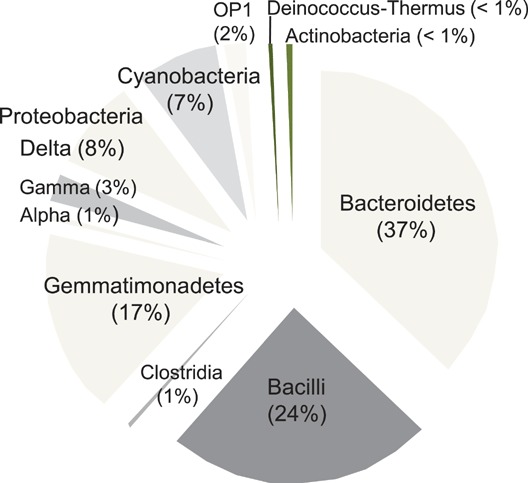
Phylogenetic distribution of clones observed in the hypersaline sediment.

## Results and Discussion

### Quantitative characterization of diversity

In this study, a total of 243 clones representing 6 bacterial divisions from the DVNP hypersaline sediment were examined. Figure 1 summarizes the phylogenetic distribution of the 16S rRNA gene sequences. The phyla Bacteroidetes, Firmicutes (classes Bacilli and Clostridia), Gemmatimonadetes, and Proteobacteria (α, γ, and δ subdivisions) predominated the bacterial community in the sediment, representing 37%, 25%, 17%, and 12% of the sequences, respectively. The least abundant sequences were represented by Cyanobacteria (7%), Actinobacteria and Deinococcus-Thermus (<1%), and OP1 (2%), a candidate division. Similar to our results, other studies of hypersaline sediments, soils, and waters, found that Bacteroidetes, Firmicutes, and/or Proteobacteria also accounted for the majority of the bacterial taxa (Mouné et al. 2003; Lefebvre et al. 2006; Mesbah et al. 2007; Hollister et al. 2010).

Within the domain Archaea, 99 clones belonging to the *Halobacteriaceae* were also examined, of which 94 sequences were found to represent 3 new genera, while the remaining 5 clone sequences were assigned to *Halorubrum*, *Haloarcula*, *Halorhabdus*, and *Halobacterium*. Ochsenreiter et al. (2002) found that most archaeal sequences recovered from DNA or cultivated strains in hypersaline waters from a solar saltern, alkaline lake, and slag heap were affiliated with *Halobacteriaceae*, specifically genera of *Halorubrum* and *Haloarcula*. In contrast, Manikandan et al. (2009) found that isolates represented by *Haloferax* and *Halorubrum* dominated solar salterns in India, with smaller numbers of *Haloarcula* and *Halogeometricum* being present.

At an evolutionary distance of 3% dissimilarity (i.e., 97% sequence identity) among the 16S rRNA genes, a total of 68 operational taxonomic units (OTUs) were obtained from the 243 bacterial clone sequences (Table 1). To estimate species richness, the ACE (abundance-based coverage), Boot, Chao1, and Jack estimators were used. At the same OTU demarcation of 3%, the respective total number of species, accounting for rare taxa not sampled, was estimated to be 110, 80, 129, and 158. These results indicate that the “rare biosphere” of low-abundance organisms is limited to several dozen species in this extreme environment, compared to marine habitats, for example, where thousands of low-abundance species are indicated (Sogin et al. 2006). Rarefaction estimates of marine diversity showed that a comparable sample of 250 organisms would yield nearly 250 OTUs, with thousands more OTUs following more extensive sampling.

**Table 1:** Estimates of taxon richness for bacterial clone libraries based on various evolutionary distance criteria for demarcating operational taxonomic units

Sequence distance criterion	Richness					Diversity index	
		
	No. of OTUs	ACE value	Boot value	Chao1 value	Jack value	Shannon	Simpson
Unique	239/243		326	28,680	478	5.476	
0.01	111	213	137	199	221	4.408	0.012
0.03	67	110	80	129	158	3.751	0.028
0.05	51	96	61	116	168	3.292	0.05
0.1	30	57	35	52	57	2.444	0.133
0.2	14	23	15	21	19	1.937	0.178

**Figure 2:**
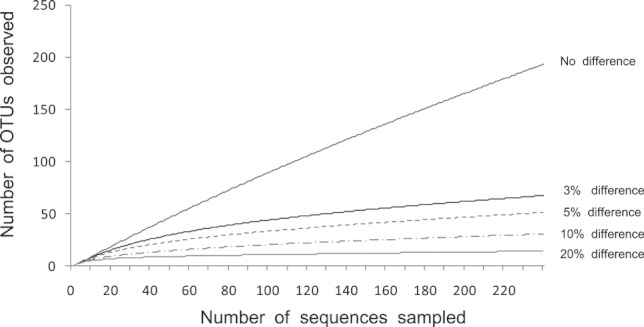
Rarefaction curves generated for bacterial 16S rRNA genes in clone libraries from the hypersaline sediment. The curves represent OTUs based on 0–20% dissimilarity.

Rarefaction estimates, using OTUs based on 0–20% distance, indicate that the clone library underestimated sediment diversity at the species (3% difference) and genus (5%) levels (Hur and Chun 2004) since none of the curves became asymptotic (Fig. 2). However, underestimation of diversity at the family (10%) and division (20%) levels was less significant since the curves did come close to reaching a plateau. The analysis of more clones would have likely resulted in the detection of additional OTUs at the species and genus levels.

### Analysis of bacterial sequences

**Figure 3:**
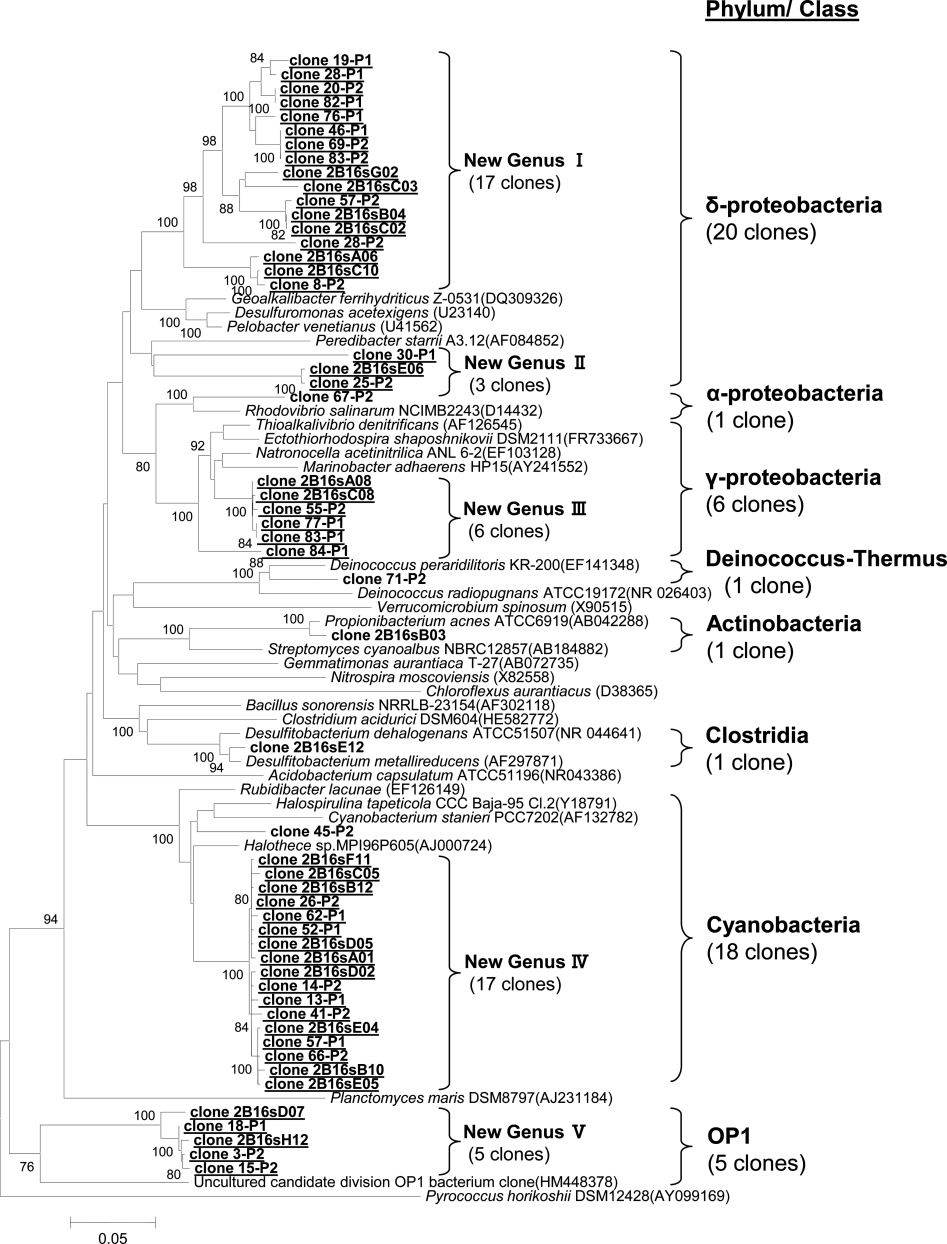
Neighbor-joining phylogenetic tree of 53 clone sequences from candidate division OP1 and divisions Proteobacteria, Actinobacteria, Deinococcus-Thermus, and Cyanobacteria from the hypersaline sediment, with reference sequences. Each clade that appears to be a previously undiscovered genus, based on 16S rRNA divergence from the nearest previously known relative, is indicated. Boostrap values >75% are noted at the branch junctions. The bar indicates a 5% estimated sequence divergence. [Correction added after first online publication on 08 April 2012: “Boostrap values >70%” has been changed to “Boostrap values >75%” in Figs 3–5, 7 and 8.]

Based on the Greengenes analysis of 16S rRNA sequence divergence of clones and isolates from their closest known relatives, we have discovered several taxa at the species and genus levels. In Figure 3, we show the discovery of two genera within δ-Proteobacteria, one genus within α-Proteobacteria, one genus within Cyanobacteria, and one genus within the OP1 candidate division.

**Figure 7:**
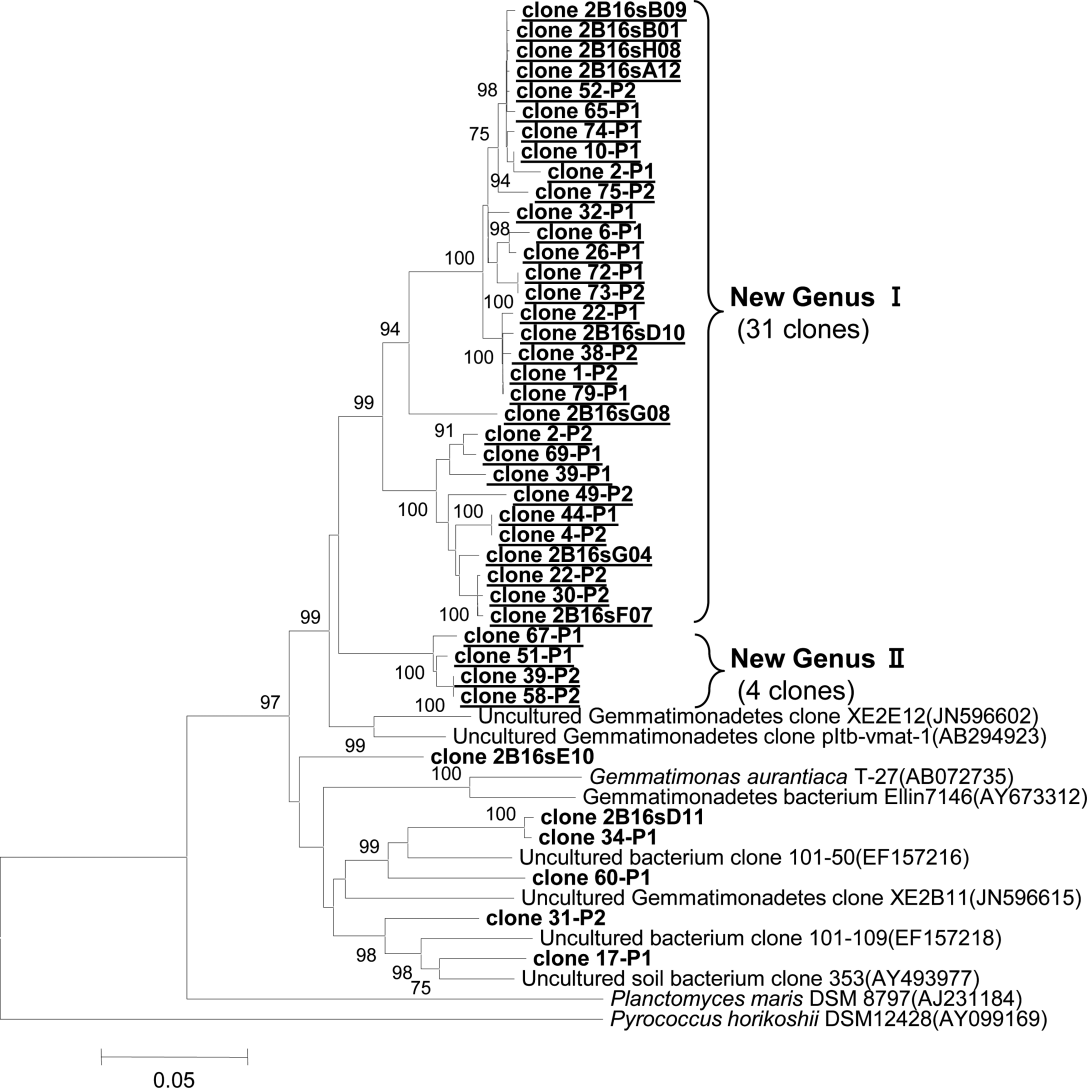
Neighbor-joining phylogenetic tree of 41 clone sequences from division Gemmatimonadetes from the hypersaline sediment. One clade of 31 clones and another clade of four clones appear to represent two previously undiscovered genera. Boostrap values >75% are noted at the branch junctions. The bar indicates a 5% estimated sequence divergence.

**Figure 8:**
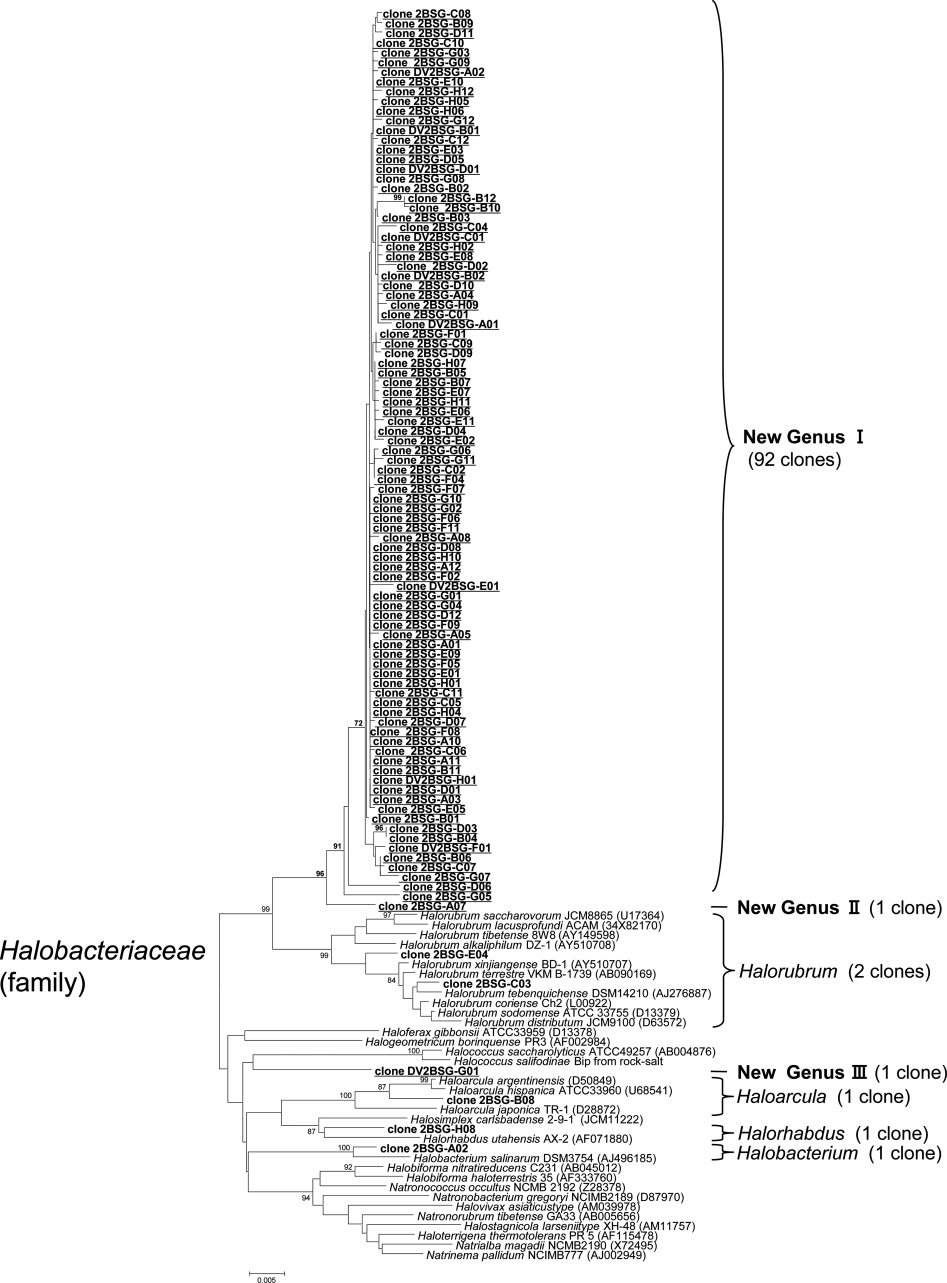
Neighbor-joining phylogenetic tree of 99 archaeal sequences obtained by cloning the 16S rRNA gene from the hypersaline sediment. Bold letters indicate clones, and underlines indicate isolates from this study. Boostrap values >75% are noted at the branch junctions. The bar indicates a 0.5% estimated sequence divergence.

**Figure 4:**
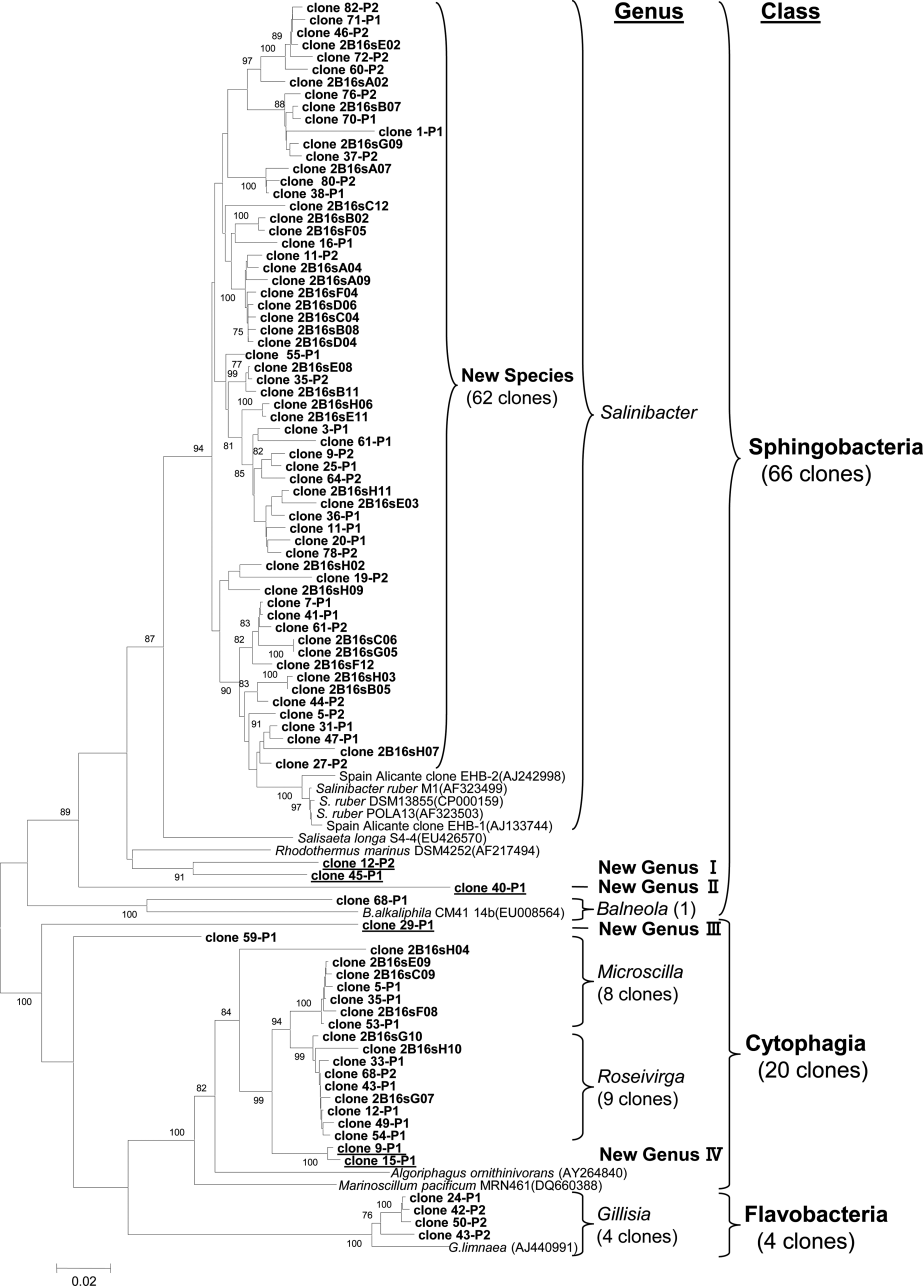
Neighbor-joining phylogenetic tree of 90 clone sequences from the division Bacteroidetes from the hypersaline sediment, with reference sequences. Two previously unknown clades are indicated as novel genera within class Sphingobacteria. Also, two clades within class Cytophagia are labeled as novel genera. Boostrap values >75% are noted at the branch junctions. The bar indicates a 2% estimated sequence divergence.

Within the phylum Bacteroidetes, the majority of the clone sequences (i.e., 74%) were at least 90% similar to *S. ruber* M31; a similar abundance of relatives of *S. ruber* was found in a previous study of DVNP evaporite crust (Bardavid et al. 2007). *Salinibacter*-like organisms have also been found at high frequencies in brines from solar salterns (Bardavid et al. 2007) and hypersaline lakes (Mutlu et al. 2008). Our 62 *Salinibacter* clones appear to represent an undiscovered species within this genus (Fig. 4). Also within the class Sphingobacteria, three clones appear to represent two newly discovered genera (Genus I and II, Fig. 4). Within the class Cytophagia, three clones appear to represent two newly discovered genera (Genus III and IV). Eight clones each within the genera *Microscilla* and *Roseivirga* appear to represent new species within these genera. Finally, within the class Flavobacteria, four clones appear to represent a new species of *Gillisia* that is most closely related to *G. limnaea*.

**Figure 5:**
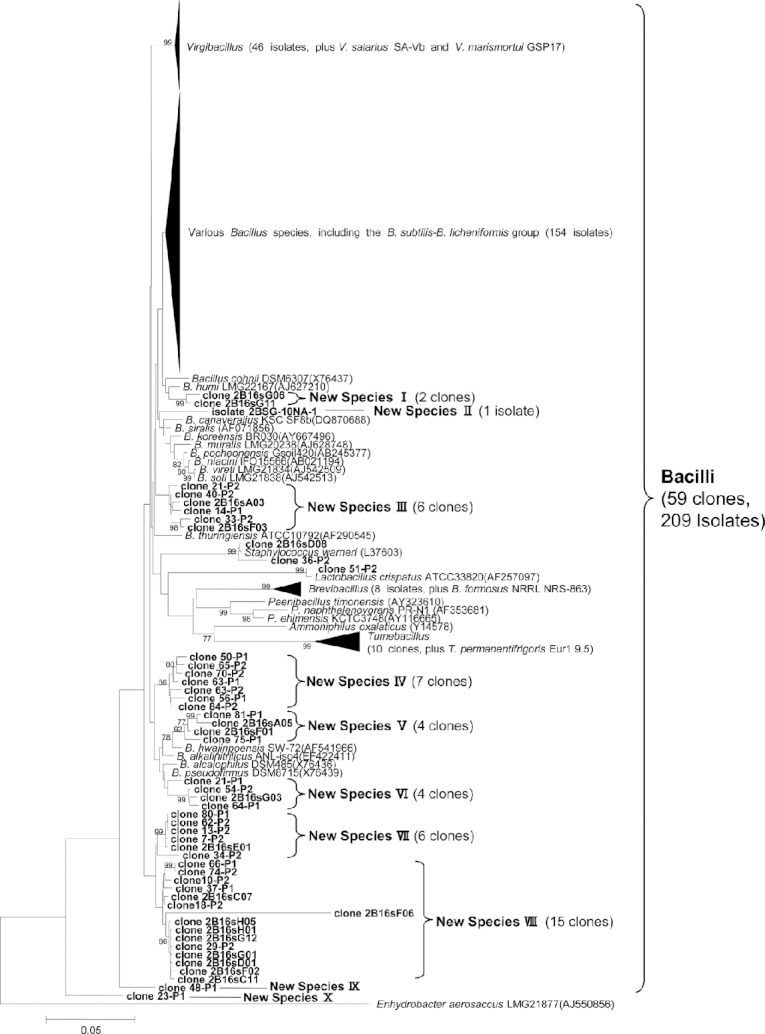
Neighbor-joining phylogenetic tree of 59 clones and 209 isolate sequences from the Bacilli from hypersaline sediment, with reference sequences. Bold letters refer to clone and isolate sequences from the hypersaline sediment. Boostrap values >75% are noted at the branch junctions. The bar indicates a 5% estimated sequence divergence.

A phylogenetic analysis revealed that the clones and isolates within the Bacilli grouped separately from one another (Fig. 5). The isolates were affiliated with three groups, *Virgibacillus* (46 isolates), various clades of *Bacillus* (154 isolates), and *Brevibacillus* (8 isolates), while the clones were dispersed into 11 other groups. As noted by other researchers (Ochsenreiter et al. 2002), the differences in results between culture-dependent and culture-independent approaches indicate that groups sampled only as isolates are likely to be at extremely low frequencies. Within Bacilli (Fig. 5), 46 of the 59 clones were tentatively identified as previously undiscovered species within the genus *Bacillus* and were closely related to *B. humi, B. cohni*, *B. thuringiensis*, *B. hwajinpoensis*, and *B. pseudofirmus*, with species IV and VII through X having no close relatives. The remaining 13 clones were affiliated with *Staphylococcus* (2), *Lactobacillus* (1), and *Tumebacillus* (10).

**Figure 6:**
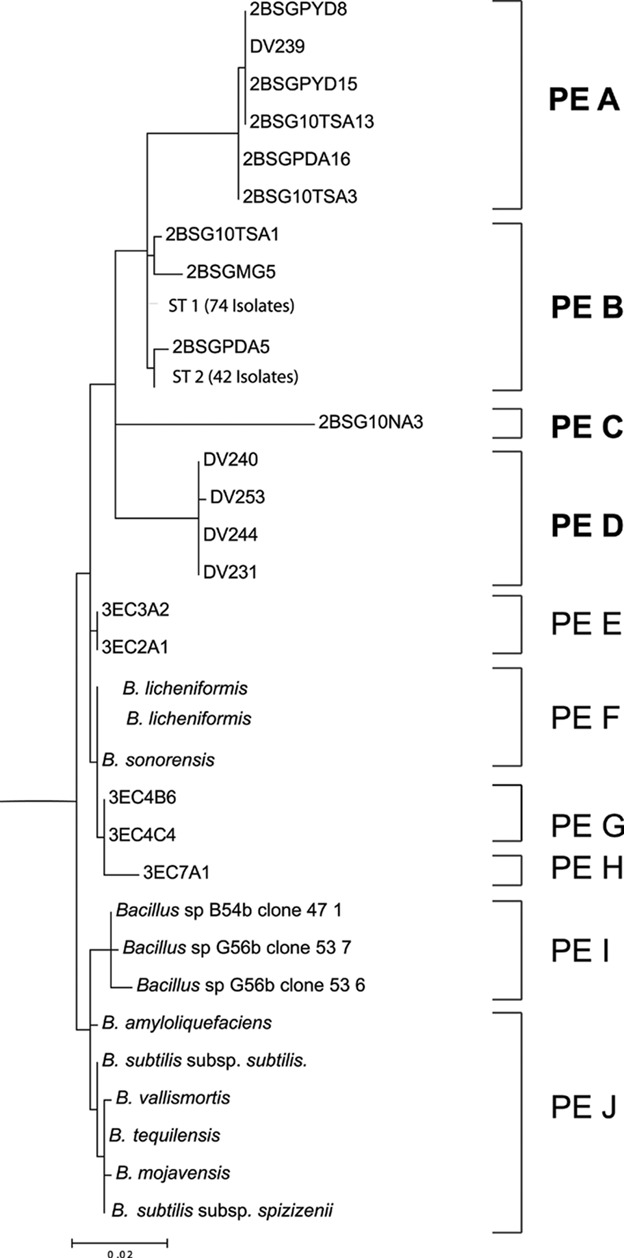
Maximum likelihood phylogeny and Ecotype Simulation analysis of 16S rRNA sequences of isolates from the *B. subtilis–B. licheniformis* clade. All isolates of this study from this clade were members of a previously unknown subclade that Ecotype Simulation demarcated into Putative Ecotypes A–D (in bold); previously studied organisms from other environments were members of Putative Ecotypes E–J. The membership of sequence types 1 and 2 are listed in Table A1. The tree is rooted by *B. halodurans* strain C-125.

The majority of the bacterial isolates from this study (130 of 209) were more closely related to the *B. subtilis–B. licheniformis* clade (Connor et al. 2010) than to any other recognized species, and these sediment isolates represent a subclade within the *B. subtilis–B. licheniformis* clade (Fig. 6). In contrast, none of the environmental clones were closely related to the *B. subtilis–B. licheniformis* clade. This indicates that the close relatives of the *B. subtilis–B. licheniformis* group are a rare minority in the hypersaline sediment sample. Because the characterized taxa closely related to *B. subtilis* are not halophilic (Gordon et al. 1973), we expect that our *B. subtilis*-related isolates are not halophilic either. We hypothesize that they and other nonhalophilic *Bacillus* isolates were washed as spores with soil from the surrounding mountains, but that in contrast to most migrants lacking a spore phase, they were able to survive without growth in the salty sediment. Thus, our isolation procedure could find them while they would be extremely rare as compared to our cultivation-free approach.

We explored the ecological diversity among the isolates most closely related to the *B. subtilis–B. licheniformis* group by employing Ecotype Simulation (Fig. 6). This algorithm is designed to identify ecologically homogeneous phylogenetic groups (ecotypes) that are ecologically distinct from other such groups (Koeppel et al. 2008). Frequently, many ecotypes are subsumed within a single-species taxon (Kopac and Cohan 2011).

A set of 130 *Bacillus* isolates formed a subclade of the *B. subtilis–B. licheniformis* clade (Fig. 6). These isolates formed four newly discovered clades that were each identified as an ecotype by Ecotype Simulation (Putative Ecotypes A–D). The ecotypes A–D contained 6, 119, 1, and 4 isolates, respectively. *Bacillus* clades previously identified as ecotypes by Ecotype Simulation have been confirmed to be ecologically distinct from one another (Wiedenbeck and Cohan 2011), and so it is reasonable to hypothesize that the newly discovered clades identified as Putative Ecotypes A–D are ecologically distinct as well. Evidence for ecological divergence among the putative ecotypes is provided by the significant tendencies of these groups to be isolated from different media (Table A2; *P* = 0.039 in Fisher's exact test). For example, all four isolates of Putative Ecotype D were isolated on marine agar (MA), while this medium yielded only a small fraction of isolates from the other putative ecotypes (17%, 17%, and 0% for Putative Ecotypes A, B, and C, respectively). The fact that the Putative Ecotype D isolates were obtained from MA suggests that they are halophilic and native members of the microbial community in the DVNP hypersaline sediment. However, they are likely in very low abundance as no sediment clones were closely related to the *Bacillus* clade.

Of the 41 clones of the Gemmatimonadetes division (Fig. 7), 2 clades (with 31 and 4 clones each) appear to represent previously undiscovered genera that are most closely related to 2 sequences extracted from a marine sponge (Montalvo and Hill 2011) and from a marine hydrothermal mat (Hirayama et al. 2007).

### Analysis of archaeal sequences

While some members of extremely halophilic Bacteria have recently been found to play a major role in hypersaline environments with salt concentrations nearing saturation (Antón et al. 2000, 2002; Bardavid et al. 2007), these environments are generally dominated by halophilic Archaea (Ventosa and Nieto 1995). We analyzed a total of 99 archaeal clones, and found that all were contained within the family *Halobacteriaceae* (Fig. 8). We discovered a pair of sister clades that each represent a previously undiscovered genus. One of these clades, with 92 sequences, is labeled as New Genus I, and its sister clade, with one sequence, is labeled New Genus II (Fig. 8). This clade of sister taxa is most closely related to *Halorubrum*. One clone, most closely related to *Halococcus*, is labeled New Genus III. In addition, we found a small number of clones classified to the species *Halorubrum tebenquichens*, and others classified to previously undiscovered species of *Halorubrum*, *Halaoarcula*, *Halorhabdus*, and *Halobacterium*. While there are currently 28 genera within the family *Halobacteriaceae*, the genus *Halorubrum* contains the largest number of species (currently 23 species). *Halorubrum* species have been isolated from hypersaline bodies of water, such as salt lakes, seas, and solar salterns (Oren 1983; Cui et al. 2007; Xu et al. 2007b).

## Conclusions

Our phylogenetic analyses of 16S rRNA gene sequences from clones and isolates revealed that the hypersaline sediment contained a variety of previously undiscovered genera, species, and ecotypes from the domains Bacteria and Archaea. We discovered 11 genera within the Bacteria and 3 genera within the Archaea, and various novel species and ecotypes. While many of the clone sequences were affiliated phylogenetically with extreme and moderate halophiles, many of the bacterial isolates may not be halophiles but instead only halotolerant, since they were cultivated on media containing low salt concentrations. Regardless, the low sequence identity of many of the clones and isolates with known reference species suggests that the hypersaline sediment contains many new and previously undescribed genera and species.

## Materials and Methods

### Sediment collection in DVNP

The geographic coordinates of the sample collection site were 36°13.063′N and 116°46.401′W. A research permit was obtained from the U.S. National Park Service prior to sample collection. Samples of hypersaline sediment, 0–5 cm below the evaporite crust, were aseptically collected using ethanol-disinfected spatulas and placed into clean, sealable plastic bags. The samples were stored in a cooler during transfer to our laboratory in Idaho, then stored at 5°C until shipped via overnight mail to the Republic of Korea. Upon receipt in Korea, the samples were stored at 5°C until processed. The hypersaline sediment had a pH of 7.65, electrical conductivity of 34.5 dS m^−1^, and chloride ion concentration of 71.3 g kg^−1^ dry sediment.

### DNA extraction, PCR, and cloning

DNA was directly extracted from 20 subsamples of the sediment using the FastDNA SPIN kit for soil according to the manufacturer's protocol (QBiogene Inc., Vista, CA). The extracted DNA was purified using FastPure DNA kit (Takara Bio Inc., Japan) and concentrated using Zymoclean gel DNA recovery kit (Zymo Research Corp., Orange, CA). The purified DNA from the 20 subsamples was then combined to increase the concentration and used to construct the clone library. The primers used to amplify the 16S rRNA genes for Bacteria and Archaea were 27F and 1492R (Lane 1991) and Ar4F and Ar958R (Jurgens et al. 2000), respectively. One microliter of DNA template was used in a 20 μL reaction mixture and PCR amplified according to the conditions as described by Lane (1991) and Jurgens et al. (2000). The thermocycler conditions were 95°C for 5 min, followed by 30 cycles of 95°C for 45 sec, 55°C for 45 sec, and 72°C for 90 sec, then a final extension step for 5 min at 72°C. Afterwards, the amplification products were purified with a QIAquick PCR purification kit (Qiagen, Valencia, CA). The purified PCR products were ligated into pUC118 HincII/BAP (Takara Bio Inc.), transformed into competent *Escherichia coli* DH4a (Invitrogen Corp., Carlsbad, CA, USA), and then plated on Luria-Bertani (LB) agar plates for selection of transformants. White recombinant transformants were selected and grown overnight at 37°C in LB medium containing 0.1 g L^−1^ ampicillin. Plasmids from *E. coli* DH4a transformants were isolated using the PureLink Quick Plasmid Miniprep kit (Invitrogen Corp.).

### Bacterial isolates

Culturable bacteria were isolated by serially diluting the sediment in sterile water, then dispensing on media plates containing R2A agar, 10% tryptic soy agar (TSA), 10% nutrient agar (NA), marine agar (MA), potato dextrose agar (PDA), peptone yeast-extract dextrose (PYD) agar, and Luria Bertani (LB) agar. The plates were incubated aerobically at 28°C for a week, after which 209 individual isolates were transferred to fresh plates and then processed for sequencing of 16S rRNA genes.

### Sequencing and phylogenetic analysis

The 16S rRNA genes from the bacterial clones and isolates were sequenced using an Applied BioSystems (Foster City, CA) model 3730xl automated DNA sequencing system. Putative chimeric sequences were identified using Bellerophon (Huber et al. 2004). 16S rRNA gene sequences were submitted to the BLAST (Basic Local Alignment Search Tool) server of the National Center for Biotechnology Information (NCBI) to determine the closest matching sequences in GenBank. The 16S rRNA sequences were aligned using the Nearest Alignment Space Termination (NAST) aligner (DeSantis et al. 2006a) and the aligned sequences were compared to the Lane mask using the Greengenes website (DeSantis et al. 2006b). Sequence matching to the Ribosomal Database Project (Cole et al. 2009) was used to find GenBank sequences representing the most closely related type strain to each clone or isolate; these type strains were included as references in the phylogeny. Using the Greengenes Automatic Taxonomic Classification algorithm (DeSantis et al. 2006b), a set of related sequences was interpreted as a novel genus (or species) if they were classified as the same genus (or species) but no previously known sequences at GenBank were classified to that genus (or species).

Phylogenetic trees were constructed using neighbor-joining (Saitou and Nei 1987), or in the case of the *Bacillus* isolates using maximum likelihood, with MEGA5 for Windows (Tamura et al. 2011). Evolutionary distances were calculated with the Kimura 2-parameter method. Bootstrap analyses of the neighbor-joining data were conducted based on 1000 samples to assess the support for inferred phylogenetic relationships.

### Quantification of diversity

The program DOTUR (Schloss and Handelsman 2005) was used to calculate taxon richness and diversity estimates. The distance matrix was obtained using the Calculate Distance Matrix algorithm located at the Greengenes website (DeSantis et al. 2006a, b).

### Ecotype Simulation analysis

The hypersaline sediment organisms more closely related to the *B. subtilis–B. licheniformis* clade than to any other taxon were identified by a BLAST search based on their 16S rRNA sequences (for species of this clade, see Connor et al. 2010). This set included 130 isolates but no environmental clones. Sequences were then aligned using the ARB software (Ludwig et al. 2004). The maximum likelihood criterion was used to reconstruct the phylogeny of this clade, based on a MEGA5 analysis of the partial sequences of 16S rRNA (Tamura et al. 2011). The phylogenetic analysis included the sequences from isolates, plus 18 type and reference strains. The tree was rooted by the sequence of *B. halodurans* strain C-125. These strains were further analyzed by Ecotype Simulation to hypothesize the demarcations of ecologically distinct clades (ecotypes), following Koeppel et al. (2008) and instructions at the Ecotype Simulation website (http://fcohan.web.wesleyan.edu).

### Nucleotide sequence accession numbers

All sequences were deposited in GenBank or DNA Databank of Japan (DDBJ) under the following accession numbers: AB533809–AB533931 and AB533933–AB534057 (bacterial clones) and AB534058–AB534156 (archaeal clones), and GQ407205–GQ407264 and AB533660–AB533808 (*Bacillus* isolates).

## Appendix

**Table A1:** The isolate membership of two 16S rRNA sequence types (ST) within Putative Ecotype A of the *B. subtilis–B. licheniformis* clade. The phylogenetic position of ST1 and ST2 is shown in Figure 6

Sequence type 1	Sequence type 2
2BSGPYD2	DV249
DV252	DV245
2BSGMG1	DV243
DV250	DV233
DV246	DV227
DV237	DV222
DV232	DV214
DV230	DV212
DV228	2BSGR2A4
DV225	2BSGR2A24
DV221	2BSGR2A21
2BSGR2A3	2BSGPYD6
2BSGR2A25	2BSGPYD19
2BSGR2A23	2BSGPYD17
2BSGR2A22	2BSGPYD10
2BSGR2A20	2BSGPDA9
2BSGR2A19	2BSGPDA8
2BSGR2A18	2BSGPDA6
2BSGR2A17	2BSGPDA3
2BSGR2A16	2BSGPDA15
2BSGR2A15	2BSGPDA11
2BSGR2A14	2BSGPDA1
2BSGR2A13	2BSGLB9
2BSGR2A12	2BSGLB7
2BSGR2A11	2BSGLB3
2BSGPYD9	2BSGLB15
2BSGPYD7	2BSGLB13
2BSGPYD5	2BSGLB10
2BSGPYD4	2BSG10TSA8
2BSGPYD3	2BSG10TSA20
2BSGPYD20	2BSG10TSA19
2BSG10NA10	2BSG10NA9
2BSG10NA11	2BSG10NA8
2BSG10NA12	2BSG10NA6
2BSG10NA17	2BSG10NA5
2BSG10NA19	2BSG10NA4
2BSG10NA20	2BSG10NA18
2BSG10NA7	2BSG10NA16
2BSG10TSA10	2BSG10NA15
2BSG10TSA11	2BSG10NA14
2BSG10TSA12	2BSG10NA13
2BSG10TSA14	DV251
2BSG10TSA15	
2BSG10TSA16	
2BSG10TSA17	
2BSG10TSA18	
2BSG10TSA2	
2BSG10TSA4	
2BSG10TSA5	
2BSG10TSA6	
2BSG10TSA9	
2BSGLB1	
2BSGLB12	
2BSGLB16	
2BSGLB17	
2BSGLB18	
2BSGLB19	
2BSGLB5	
2BSGLB8	
2BSGPDA12	
2BSGPDA13	
2BSGPDA14	
2BSGPDA17	
2BSGPDA18	
2BSGPDA19	
2BSGPDA4	
2BSGPDA7	
2BSGPYD16	
2BSGPYD18	
2BSGPYD14	
2BSGPYD13	
2BSGPYD12	
2BSGPYD11	
2BSGPYD1	

**Table A2:** The media used to isolate strains of the four putative ecotypes within the *B. subtilis–B. licheniformis* clade (in bold), as well as from other taxa. A Fisher's exact contingency test was run on the three putative ecotypes of the *B. subtilis–B. licheniformis* clade with greater than one isolate (i.e., excluding C), with the three peptone-containing, nonsaline media pooled (i.e., the first three media). This yielded a significant difference across ecotypes in their tendencies to be isolated by the various media (*P* = 0.039)

Medium	Putative ecotype A	Putative ecotype B	Putative ecotype C	Putative ecotype D	*Virgibacillus*	*Brevibacillus*	Bacilli new species II
10% nutrient agar	**0**	**17**	**1**	**0**	0	1	1
Luria broth agar	**0**	**14**	**0**	**0**	5	2	0
Peptone yeast extract dextrose agar (PYD)	**2**	**18**	**0**	**0**	0	0	0
Potato dextrose agar (PDA)	**1**	**16**	**0**	**0**	0	0	0
R2A agar	**0**	**17**	**0**	**0**	0	5	0
10% Tryptic soy broth agar	**2**	**17**	**0**	**0**	0	1	0
Marine agar	**1**	**20**	**0**	**4**	41	0	0
